# Plasm Metabolomics Study in Pulmonary Metastatic Carcinoma

**DOI:** 10.1155/2022/9460019

**Published:** 2022-08-21

**Authors:** Zixu Liu, Ling Wang, Minjun Du, Yicheng Liang, Mei Liang, Zhili Li, Yushun Gao

**Affiliations:** ^1^Department of Thoracic Surgery, National Cancer Center/National Clinical Research Center for Cancer/Cancer Hospital, Chinese Academy of Medical Sciences and Peking Union Medical College, Beijing, China; ^2^Department of Thoracic Surgery, National Cancer Center/National Clinical Research Center for Cancer/Hebei Cancer Hospital, Langfang, China; ^3^Department of Hematology, Beijing Chuiyangliu Hospital, Beijing, China; ^4^Department of Biophysics and Structural Biology, Institute of Basic Medical Sciences, Chinese Academy of Medical Sciences & School of Basic Medicine, Peking Union Medical College, Beijing, China

## Abstract

**Background:**

The lung is one of the most common metastatic sites of malignant tumors. Early detection of pulmonary metastatic carcinoma can effectively reduce relative cancer mortality. Human metabolomics is a qualitative and quantitative study of low-molecular metabolites in the body. By studying the plasm metabolomics of patients with pulmonary metastatic carcinoma or other lung diseases, we can find the difference in plasm levels of low-molecular metabolites among them. These metabolites have the potential to become biomarkers of lung metastases.

**Methods:**

Patients with pulmonary nodules admitted to our department from February 1, 2019, to May 31, 2019, were collected. According to the postoperative pathological results, they were divided into three groups: pulmonary metastatic carcinoma (PMC), benign pulmonary nodules (BPN), and primary lung cancer (PLC). Moreover, healthy people who underwent physical examination were enrolled as the healthy population group (HPG) during the same period. On the one hand, to study lung metastases screening in healthy people, PMC was compared with HPG. The multivariate statistical analysis method was used to find the significant low-molecular metabolites between the two groups, and their discriminating ability was verified by the ROC curve. On the other hand, from the perspective of differential diagnosis of lung metastases, three groups with different pulmonary lesions (PMC, BPN, and PLC) were compared as a whole, and then the other two groups were compared with PMC, respectively. The main low-molecular metabolites were selected, and their discriminating ability was verified.

**Results:**

In terms of lung metastases screening for healthy people, four significant low-molecular metabolites were found by comparison of PMC and HPG. They were O-arachidonoyl ethanolamine, adrenoyl ethanolamide, tricin 7-diglucuronoside, and p-coumaroyl vitisin A. In terms of the differential diagnosis of pulmonary nodules, the significant low-molecular metabolites selected by the comparison of the three groups as a whole were anabasine, octanoylcarnitine, 2-methoxyestrone, retinol, decanoylcarnitine, calcitroic acid, glycogen, and austalide L. For the comparison of PMC and BPN, L-tyrosine, indoleacrylic acid, and lysoPC (16 : 0) were selected, while L-octanoylcarnitine, retinol, and decanoylcarnitine were selected for the comparison of PMC and PLC. Their AUCs of ROC are all greater than 0.80. It indicates that these substances have a strong ability to differentiate between pulmonary metastatic carcinoma and other pulmonary nodule lesions.

**Conclusion:**

Through the research of plasm metabolomics, it is possible to effectively detect the changes in some low-molecular metabolites among primary lung cancer, pulmonary metastatic carcinoma, and benign pulmonary nodule patients and healthy people. These significant metabolites have the potential to be biomarkers for screening and differential diagnosis of lung metastases.

## 1. Introduction

Pulmonary metastatic carcinoma is one of the most common sites of distant metastasis in many advanced cancers. Early detection, accurate diagnosis, and appropriate treatment are the effective methods to reduce the mortality of pulmonary metastases. At present, the main screening method for pulmonary metastatic carcinoma is the chest low-dose CT scan. Compared with chest X-rays, it has the advantage of higher resolution and can find smaller sized pulmonary nodules, which is conducive to early detection. However, the CT scan also has a certain false positive rate, as well as overdiagnosis, higher cost, stronger radiation, and other shortcomings. Meanwhile, the differential diagnosis of pulmonary nodules by chest CT is sometimes not satisfactory. Different pathological types such as primary lung cancer, pulmonary metastatic carcinoma, and benign pulmonary nodules are often confused with each other in clinical practice and cannot be clearly diagnosed. Tumor markers commonly used for lung cancer include carcinoembryonic antigen (CEA), cytokeratin 19 (Cyfra21-1), neuron-specific enolase (NSE), gastrin-releasing peptide precursor (pro-GRP), and squamous cell carcinoma antigens (SCAA). Nevertheless, they all have the disadvantage of low sensitivity and specificity for lung metastases, resulting in their limited role in clinical application.

Nowadays, blood tumor markers for lung cancer are the focus point of many studies. Various kinds of blood substances have been used as researching subjects, such as circulating tumor cells (CTC), circulating tumor DNA (ctDNA), micro-RNA (miRNA), autoantibodies (AAbs), plasma proteins, and low-molecular metabolites. Metabolomics is an important complement to genomics, transcriptomics, and proteomics. Metabolic change is located in the downstream of DNA, RNA, and protein changes. Through qualitative and quantitative studies on low-molecular metabolites, the functional status and pathophysiological changes of the body can be understood more directly and deeply. Metabolomics is divided into targeted metabolomics and nontargeted metabolomics generally. Nontargeted metabolomics is the overall analysis of the metabolome to identify different metabolites, which is used as a preliminary selection of biomarkers. Targeted metabolomics applies a uniform standard to quantify metabolites, which result is more accurate and reliable than nontargeted metabolomics.

Metabolomics has been widely used to study a variety of diseases in the human body, including mental disorders [1], type 2 diabetes mellitus [2], autoimmune diseases [3], and malignant tumors [4]. Metabolomics research of cancer in various organs has been reported [5–8] in recent years. Previous metabolomics research on lung cancer has focused on some certain areas, such as the difference between lung cancer and healthy people [9, 10] or chronic obstructive pulmonary disease (COPD) [11], the distinction among pathological types or stages of lung cancer [12–14], the monitoring of drug effects [15, 16], and the judgment of cancer treatment prognosis [17]. These studies had some shortcomings in common, such as the same kind of research subjects, incomplete research scope, and lack of verification and repetitiveness. At present, lung metastatic cancer is an area that lacks in-depth research.

The purpose of this study was to select some significant low-molecular metabolites in plasm as potential biomarkers, by the comparison of plasm samples from pulmonary metastatic carcinoma, benign pulmonary nodules, primary lung cancer, and healthy people. These selected metabolites may play a vital role in clinical screening of lung metastases and differential diagnosis of pulmonary nodules in the future. A preprint has previously been published [18].

## 2. Materials and Methods

### 2.1. Participants

**Figure 1:**
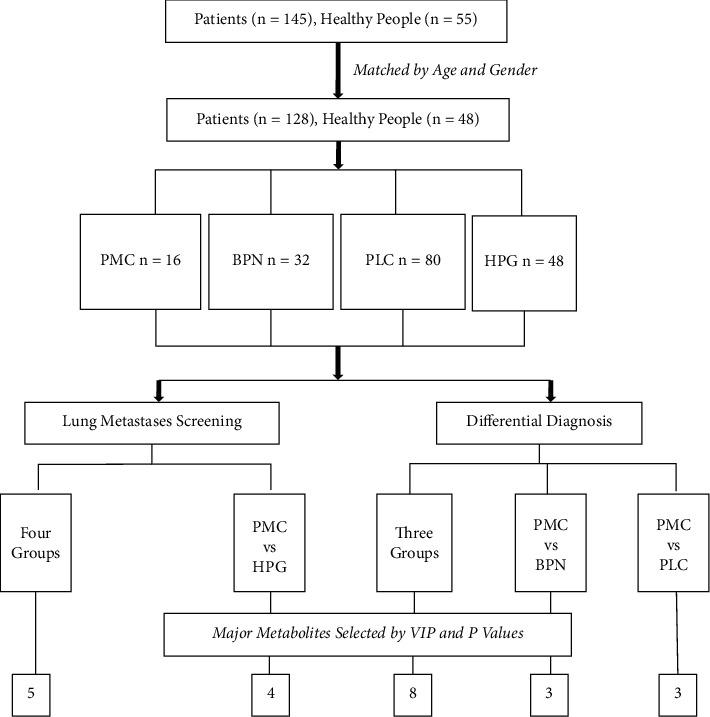
Flowchart of the study.

This study was approved by the Ethics Committee of the National Cancer Center, Chinese Academy of Medical Sciences, and its number is 19/223-2007. The flowchart of this study is shown in Figure 1. A total of 145 patients with pulmonary nodules admitted to our department from February 1, 2019, to May 31, 2019, were collected, and 128 people were selected according to the gender- and age-matching results. They were divided into pulmonary metastatic carcinoma (PMC), benign pulmonary nodules (BPN), and primary lung cancer (PLC) by postoperative pathology. A total of 48 healthy people who underwent physical examination during the same period were enrolled as the healthy population group (HPG) in this study. At last, there were 16 patients in PMC, 32 patients in BPN, 80 patients in PLC, and 48 people in HPG. The general characteristics of these subjects are shown in Table 1.

On the one hand, to study the role of low-molecular metabolites in pulmonary metastases screening, we firstly compared pulmonary metastatic carcinoma (PMC) with the healthy population group (HPG), benign pulmonary nodule (BPN), and primary lung cancer (PLC) as a whole and then compared PMC with HPG solely. On the other hand, to study the role of low-molecular metabolites in the differential diagnosis of lung metastases, we compared PMC, BPN, and PLC on the whole and then compared PMC with BPN and PLC, respectively.

### 2.2. Sample Collection and Preparation

To avoid the effects of food and time on low-molecular metabolites, blood samples of all subjects were taken under the fasting state in the morning. The blood samples were immediately placed in an EDTA anticoagulation tube and then centrifuged at 4°C, 3000*g* for 5 minutes. Then the plasm was taken and stored in −80°C. After thawing the plasm samples at 4°C, 50 *μ*l of plasm was taken, and 950 *μ*l of methanol/acetonitrile (2/3, v/v) solution was added and then vortexed for 1 minute. After standing at 4°C for 24 hours, the sample was centrifuged at 19,000*g* for 30 minutes. Then 50 *μ*l of the supernatant was taken, and 250 *μ*l of dichloromethane and 500 *μ*l of ultrapure water were added and mixed. After vortexing for 30 seconds, the sample was centrifuged at 1250*g* for 6 minutes. Then 75 *μ*l of the supernatant was transferred to a glass bottle and allowed to dry naturally at room temperature. The dried sample was redissolved by adding 150 *μ*l of 50% methanol solution.

### 2.3. Instrumental Test

The plasma metabolites profiling was performed on the platform of Quadrupole Time of Flight tandem mass spectrometry. Mass spectrometer is an instrument for substance separation and detection by measuring the mass-to-charge ratio (m/z) of the tested sample ions. The samples were first ionized, and then the mass spectra were obtained by separating the ions according to different motion behavior of different ions in the electric field. Finally, the qualitative and quantitative results of the samples are obtained. For every 20 normal samples, a quality control (QC) sample was inserted to ensure the quality of the experiment. The QC samples were mixed from four different types of plasm samples. A total of 8 QC samples were tested in the whole experiment. In addition, 4 blank samples were tested. The blank samples can be used to check the residues of the substance during the test. During the data processing, the experimental samples and the blank samples can be compared to select the unique or abundant low-molecular metabolites with research value.

### 2.4. Data Processing and Annotation of Metabolites

The original data of each sample were imported into the DataAnalysis 4.4 software, and the time period of 0–0.4 min when the main peak appeared in the detection process was taken to obtain about 900 characteristic peaks with different molecular weights between 50 and 1000 Da. The characteristic peaks with absolute intensity less than 1000 were removed, and then the deconvolution process was performed to combine the isotopes of the same metabolite. Finally, 180 features of different samples were obtained, and then 155 representative features were selected for identification of low-molecular metabolites. Different m/z values of the features were entered into the Human Metabolome Database (HMDB) to obtain the corresponding molecular formula, and then the IsotopePattern software was used to get the isotopic abundance of this molecular, which was compared with the isotopic abundance of the corresponding characteristic peaks displayed in the DataAnalysis 4.4 software. The names of the selected low-molecular metabolites can be determined by comprehensive judgments such as the accordance degree of isotope abundance, the difference of molecular weight, the types of inducers, the generation mode, and the existence range of metabolites. Human Metabolome Database (HMDB), PubChem, and MassBank databases were searched to assist with metabolite identification. Analyte mass accuracy (in the range of 0 to 10 ppm), retention time, isotope pattern, and tandem mass spectrometry fragmentation pattern were used for final determination of the selected metabolites.

A computer program was designed by Python language to summarize the absolute intensity values of 155 low-molecular metabolites in four groups of 176 samples. According to the 80% rule, the metabolites existing in at least 140 samples were retained, and as a result, a total of 80 low-molecular metabolites were obtained. Although some low-molecular metabolites were present in 80% or above of the samples, they still do not exist in a minority of samples. The intensity value of the metabolite in such samples was set to half of the lowest absolute intensity value of the same metabolite in other samples.

### 2.5. Statistical Analysis

The data of each comparison group were normalized by MetaboAnalyst 4.0, and preliminary analysis was performed. Normalized data were imported into the Simca 14.1 software for multivariate data statistical analysis, including principal component analysis (PCA), partial least squares discriminant analysis (PLS-DA), orthogonal partial least squares discriminant analysis (OPLS-DA), etc. Before performing multivariate data statistical analysis, all data were log-transformed and Par (Pareto variance scaling)-formatted to obtain more reliable and intuitive results. PCA can reflect the overall metabolic difference among groups and the degree of variability between samples within the same group. OPLS-DA can filter signals that are irrelevant to the model classification and obtain OPLS-DA models, so as to clarify the relevant low-molecular metabolites with significant difference. The quality of the model was tested by cross-validation, and the validity of the model was judged by the R2X and Q2 (representing the model's interpretable variables and the predictability, respectively). The validity of the model is further tested by permutation experiments.

### 2.6. Biomarkers Selection

The difference of the comparison between two groups was judged by indicators including variable importance in projection (VIP), *p* value, and fold change (FC). In order to select the proper biomarkers, we first calculate the VIP value of metabolites that lead to the difference between two groups in the OPLS-DA model. It is generally considered that metabolites with VIP >1 have analytical significance. These low-molecular metabolites were statistically compared by SPSS 19.0 software. Because the data between groups did not conform to the normal distribution, the nonparametric test was used (comparisons between two groups were performed by the Mann-Whitney *U* test; comparisons among three or four groups were performed by the median test). Since there were more than one metabolites with VIP >1 in each comparison, and the statistical difference between the groups was tested more than once, the Bonferroni method was used to correct the threshold of *p* value. In each comparison between the groups, several most representative low-molecular metabolites were selected as biomarkers by the VIP and *p* value, and their diagnostic sensitivity and specificity were judged by the ROC curve.

## 3. Results

### 3.1. Comparison of General Characteristics

After matching of age and gender, a total of 176 people participated in the study who are from four groups: pulmonary metastatic carcinoma group (PMC, *n* = 16), benign pulmonary nodule group (BPN, *n* = 32), primary lung cancer group (PLC, *n* = 80), and healthy population group (HPG, *n* = 48). The detailed clinicopathological characteristics of the patients were in Table 1, including age, gender, smoking history, comorbidity, tumor site, and pathological types. There were no significant differences in the comparison among these groups (*p* > 0.05).

The primary source of pulmonary metastatic carcinoma were rectal cancer (*n* = 7), colon cancer (*n* = 4), lower limb osteosarcoma (*n* = 1), nasal cancer (*n* = 1), breast cancer (*n* = 2), and liver cancer (*n* = 1). Postoperative pathological types in the benign pulmonary nodule group included lymphadenopathy (*n* = 11), tuberculous granulomatosis (*n* = 4), sclerosing alveolar cell tumor (*n* = 2), organizing pneumonia (*n* = 5), hamartoma (*n* = 3), leiomyoma (*n* = 2), solitary fibroma (*n* = 1), fungal infection (*n* = 1), atypical adenomatous hyperplasia (*n* = 2), and epithelioid hemangioendothelioma (*n* = 1).

### 3.2. Lung Metastases Screening

Firstly, we compare the four groups as a whole and obtain the overall difference among them. Then pulmonary metastatic carcinoma was compared with the health population group. The differences in plasm metabolic profiles between them were analyzed, and the low-molecular metabolites that cause these differences were selected.

#### 3.2.1. Overall Comparison of the Four Groups

**Figure 2:**
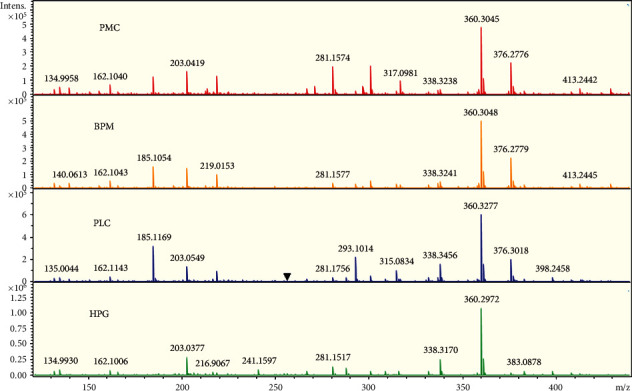
From top to bottom are the typical peak spectra of samples from PMC, BPN, PLC, and HPG, respectively. The *X*-axis represents the m/z value, which can be understood as the molecular weight of the substance; different m/z values represent the relevant low-molecular metabolites; the *Y*-axis represents the absolute intensity value of the substance contained in the sample. Comparing the four groups visually, it can be seen that most of the low-molecular metabolites are present in the four groups, but the absolute intensity values are different in various samples. There are some low-molecular metabolites that are unique to a certain sample or not present in a certain sample. The main reason is that the absolute intensity value is extremely low, causing the peak height too low to be identified in the figure.

The raw data of each sample in the four groups were imported into the DataAnalysis 4.4 software to obtain the corresponding peak spectrum. The typical peak spectra of samples from the four groups are shown in Figure 2. The information of the low-molecular metabolites contained in each sample was listed. After a series of procedures including absolute intensity values filtrating, deconvolution, and comprehensive identification, 78 low-molecular metabolites were identified at last. The other two metabolites could not be interpreted. The identified low-molecular metabolites involved a variety of material types, including amino acids, vitamins, sugars, choline, organic acids, triglycerides, cholesterol, and other substances.

**Figure 3:**
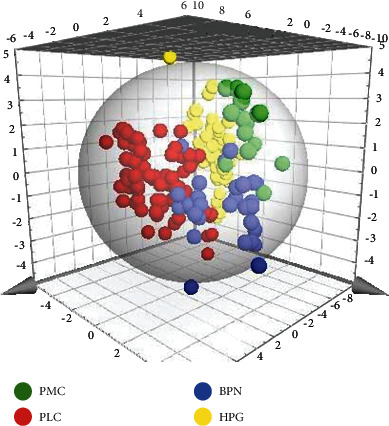
Three-dimensional graphic is obtained after statistical analysis of multivariate variables in four groups. It can be seen that the spatial distribution of samples in different groups have a clear trend of dispersion. The spatial distribution of samples in the same group has a trend of aggregation, indicating that the level of low-molecular metabolites in different groups is apparently different.

**Figure 4:**
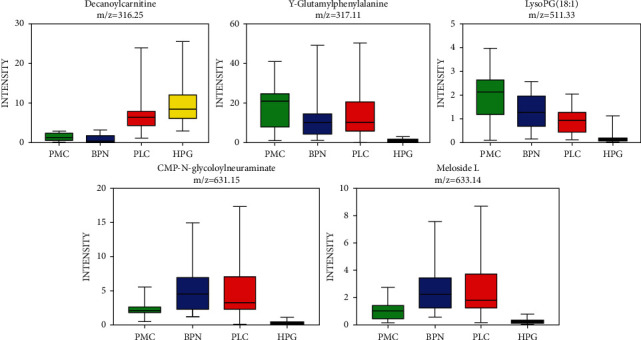
After comparison among the four groups, five low-molecular metabolites were selected, namely decanoylcarnitine, *γ*-glutamylphenylalanine, lysophosphatidyl glycerol (18 : 1), CMP-N-glycoloylneuraminate, and meloside (L.) The intensity values of these five metabolites in each group can be intuitively reflected by box plots.

The data of four groups were normalized by MetaboAnalyst 4.0. The normalized data were imported into the Simca 14.1 software for multivariate data statistical analysis. As shown in Figure 3, the spatial distribution of all samples in different groups had a clear trend of dispersion. However, the distribution of the samples in the same group had a trend of aggregation, which indicated that the levels of low-molecular metabolites in different groups were obviously different overall. After calculating the VIP values of all metabolites that cause the difference of distribution in each group, it was found that there were 28 low-molecular metabolites with VIP >1. The intensity values of these metabolites were calculated in the nonparametric tests by the SPSS 19.0 software. A total of 22 metabolites were less than the *p* value corrected by the Bonferroni method. According to the VIP value and *p* value, five low-molecular metabolites were selected, including decanoylcarnitine, glutamylphenylalanine, lysophosphatidyl glycerol (18 : 1), CMP-N-glycoloylneuraminate, and meloside L. The intensity values of these five metabolites in each group can be intuitively reflected by box plots, as shown in Figure 4. The intensity values of each low-molecular metabolite were compared among the four groups. After nonparametric tests, the *p* values obtained were all less than 0.001, indicating that the level of the five metabolites in the four groups was significantly different.

#### 3.2.2. Comparison of the Healthy Group and the Pulmonary Metastases

**Figure 5:**
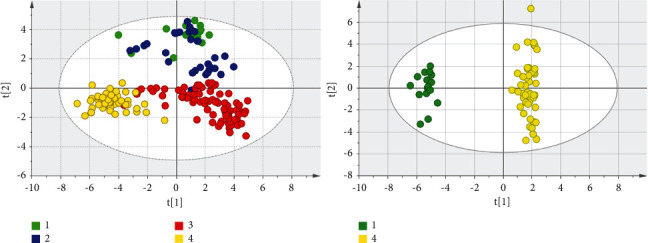
Score scatter plots by OPLS-DA analysis are shown. The number 1, 2, 3, and 4 in the figures represent PMC, BPN, PLC, and HPG, respectively. It can be seen that there is obvious difference in the distribution of the four groups, and the difference in the distribution between the pulmonary metastatic carcinoma and the healthy group can be seen clearly.

The pulmonary metastatic carcinoma (PMC) was compared with HPG solely. After multivariate data statistical analysis, the score scatter plot of this comparison was obtained, as shown in Figure 5. The clear separation of the two groups can be seen. After the nonparametric tests, there were 29 low-molecular metabolites with VIP >1 and *p* < 0.001. The major low-molecular metabolites were selected according to the fold change, VIP value, and *p* value in each comparison, as shown in Table 2. The fold change is the ratio of the absolute intensity values of the metabolites in the comparison between pulmonary metastases and healthy groups. The value of greater than one indicates a higher level of the low-molecular metabolite in the corresponding pulmonary nodule group than the healthy group.

**Figure 6:**
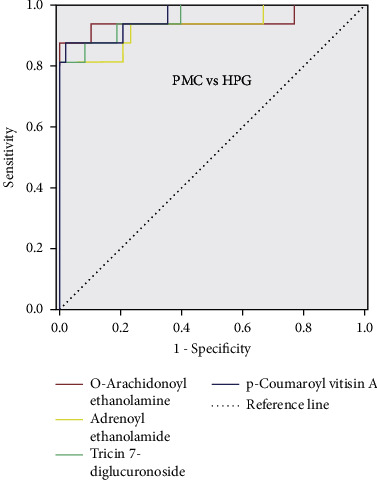
ROC curves of the major low-molecular metabolites in the comparison between the pulmonary metastatic carcinoma and healthy people.

The major low-molecular metabolites that caused difference between PMC and HPG were o-arachidonoyl ethanolamine, adrenoyl ethanolamide, tricin 7-diglucuronoside, and p-coumaroyl vitisin A. In order to test the ability of these metabolites to discriminate between the healthy people and the pulmonary metastatic carcinoma, these major low-molecular metabolites were drawn into ROC curves according to the comparison, as shown in Figure 6. The area under the curve (AUC) of every low-molecular metabolite was greater than 0.9, indicating that these low-molecular metabolites all have a high discriminating ability. The critical point of each ROC curve and the sensitivity and specificity corresponding to the critical point are shown in Table 3. According to the calculation method of the Yoden index, Yoden index = sensitivity + specificity − 1, the Yoden index of these four substances were 0.875, 0.813, 0.813, and 0.854, respectively. The higher the Yoden index, the better the screening effect.

### 3.3. Differential Diagnosis of Pulmonary Metastases

According to the pathology, the common pulmonary nodules were mainly divided into three types: primary lung cancer, benign pulmonary nodules, and pulmonary metastatic carcinoma. To help determine the nature of lung nodules before surgery, the three types of pulmonary nodules were compared by the means of plasm metabolomics.

#### 3.3.1. Overall Comparison of the Three Groups

**Figure 7:**
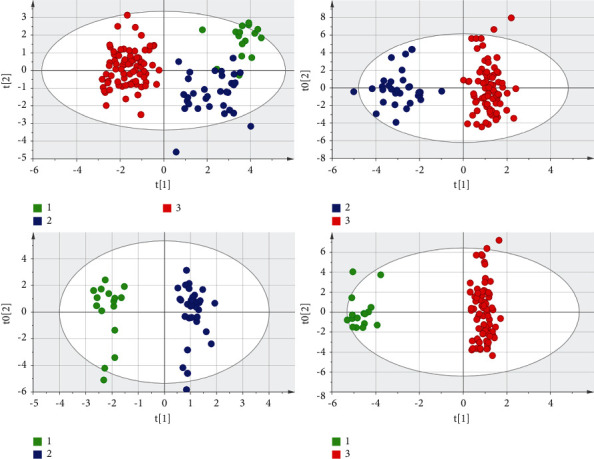
The score scatter plots of the overall comparison of the three pulmonary nodule groups and the pairwise comparisons are shown. It can be seen intuitively that the differences among the three groups are obvious. The number 1, 2, and 3 in the figures represent PMC, BPN, and PLC, respectively.

**Figure 8:**
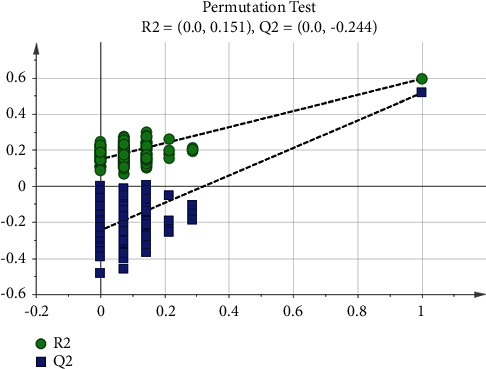
Permutation test of the three pulmonary nodule groups. The number of tests is 200; the *X*-axis represents the correlation between the random group *Y* and the original group *Y*, and the *Y*-axis represents the scores of R2 and Q2. The rightmost is the real value, and the left is the simulated value. The Q2 value of all the blue on the left is lower than the original point on the right, the R2 value of all the green on the left is lower than the original point on the right, and the intercept of the regression line of Q2 is <0.05.

**Figure 9:**
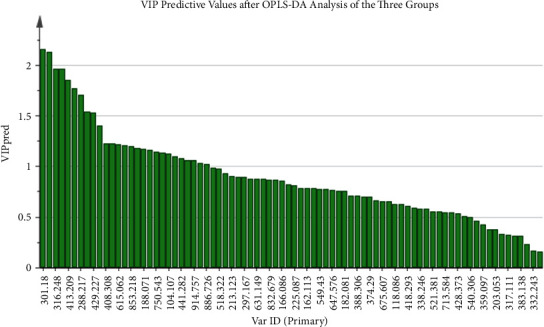
After OPLS-DA analysis, the predicted values of all low-molecular metabolites are calculated in the overall comparison of the three pulmonary nodule groups. The larger the VIP value is, the greater the role of identifying the difference among groups is.

First the PMC, BPN, and PLC were compared as a whole. After the OPLS-DA analysis, the score scatter plots of the three groups were obtained, as shown in Figure 7. The distribution among the groups was dispersive, and the distribution within the same group was concentrated, indicating that the levels of low-molecular metabolites in the three groups were different. The validity of the model was further tested by the permutation test. The number of tests was set to 200. The test result showed that the validity was good (Figure 8). VIP and *p* values of low-molecular metabolites were calculated in the overall comparison of the three groups, and there were 27 metabolites with VIP value >1 and *p* value <0.001 (Figure 9). The major low-molecular metabolites were selected based on VIP and *p* values: anabasine, octanoylcarnitine, 2-methoxyestrone, retinol, decanoylcarnitine, calcitroic acid, glycogen, and austalide L. The metabolic pathways involved include lipid metabolism (glycerol phospholipid metabolism, linoleic acid metabolism, *α*-linolenic acid metabolism, arachidonic acid metabolism, and steroid hormone synthesis), amino acid metabolism (glycine, serine, and threonine metabolism), and so on.

#### 3.3.2. Comparison of the Lung Metastases and the Other Pulmonary Nodule Groups

**Figure 10:**
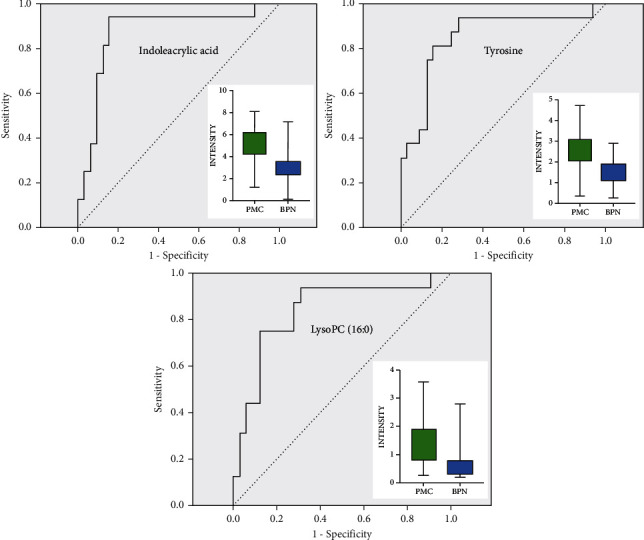
ROC curves and box plots of three major low-molecular metabolites in the comparison between PMC and BPN.

**Figure 11:**
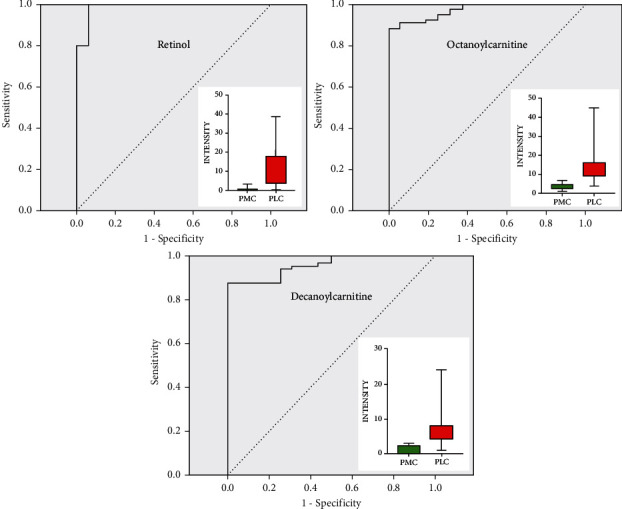
ROC curves and box plots of three major low-molecular metabolites in the comparison between PMC and PLC.

The PMC were compared with BNP and PLC, respectively. In the first place, PMC and BPN were compared. Tyrosine, indoleacrylic acid, and lysoPC (16 : 0) were selected. Their ROC curves and box plots were shown in Figure 10. The metabolic pathways involved in these metabolites are amino acid synthesis and metabolism (phenylalanine, tyrosine, and tryptophan), lipid synthesis and metabolism (glycerol phospholipids and steroids), aminoacyl biosynthesis, ubiquinone, and other terpene quinone biosynthesis, etc. In the second place, the PMC was compared with PLC. The score scatter plot after OPLS-DA analysis was shown in Figure 8, and the difference between the two groups was obvious. We calculated the VIP values of the low-molecular metabolites that cause the difference. There were 18 metabolites with VIP value >1 and *p* value <0.001. The main low-molecular metabolites selected were octanoylcarnitine, retinol, and decanoylcarnitine. As shown in Figure 11, the difference of the three metabolites between the two groups can be seen directly in the box plot. ROC curves were drawn for these metabolites, and the areas under the curve (AUCs of ROC) of the three metabolites were 0.973, 0.988, and 0.956, respectively. The critical points for obtaining the best sensitivity and specificity were 6.51, 0.99, and 3.01, accordingly. Detailed information was shown in Table 2.

## 4. Discussion

According to the previous study [19], gender and age may cause changes in plasm metabolic profiles. To avoid such influence of these factors, from 145 patients with pulmonary nodules admitted to our hospital and 55 healthy people in the medical center, only 128 patients and 48 healthy volunteers were selected as research subjects according to the gender- and age-matching principle. In previous metabolomics studies on the lung, it was common to compare primary lung cancer with healthy people [20]. A small number of literatures had reported benign pulmonary nodules or chronic obstructive pulmonary disease compared with primary lung cancer [21, 22]. However, there were rare reports on pulmonary metastatic carcinoma until now, no matter what sources of the pulmonary samples, such as lung tissue, sputum, bronchial lavage fluid, etc. This study firstly reported the plasma metabolites characteristics of lung metastases and summarized the difference of among three common pulmonary nodules and healthy people.

The sixteen people in the pulmonary metastatic cancer group in this study had a wide range of primary carcinoma sources, including colorectal cancer, liver cancer, breast cancer, nasal cancer, and lower extremity osteosarcoma. Therefore, the score scatter plot after OPLS-DA analysis showed that most points were aggregated, but there were several points that were relatively discrete, as shown in Figure 5. Pathological types of the 32 samples from the benign pulmonary nodule group included lymphadenitis, tuberculous granuloma, sclerosing alveolar cell tumor, organizing pneumonia, hamartoma, leiomyoma, solitary fibroid, fungal infection, atypical adenomatous hyperplasia, and epithelioid hemangioendothelioma. As a result, the points of its score scatter plot were more discrete, but when compared with other groups, it mainly showed obvious aggregation (Figure 5). For the score scatter plots of the primary lung cancer group and the healthy person group, except for several points, the most were clustered together, indicating that these samples had better consistency and the results were more reliable.

Through the statistical analysis of the multivariate data of the Simca 14.1 software, the OPLS-DA models obtained in the comparisons among the groups were shown in Figures 5 and 7, and their characteristic parameters were shown in Table 4. R2X and R2Y represent the percentage of the *X* and *Y* matrix information that the model can interpret, respectively, and Q2 represents the predictive ability of the model obtained through cross-validation calculation. A good model generally includes several conditions: R2Y is always greater than Q2; the higher the values of R2 and Q2, the better; the difference between R2Y and Q2 is not too big, preferably less than 0.3; R2X is above 0.5. According to Table 4, the OPLS-DA model of each comparison among groups was a good model that met the conditions.

Screening for lung metastases relies on a chest low-dose CT scan and tumor markers in the present, but a CT scan may show false-positive results. Tumor markers for lung cancer such as CEA, Cyfra21-1, NSE, and pro-GRP, due to their low sensitivity and specificity, have limited clinical application value. In order to find biomarkers with high sensitivity and specificity, we took plasm low-molecular metabolites as the research subject, comparing pulmonary metastatic cancer with the primary lung cancer, pulmonary benign nodule, and healthy people as a whole. Five major low-molecular metabolites were selected, namely decanoylcarnitine, *γ*-glutamylphenylalanine, lysophosphatidyl glycerol (18 : 1), CMP-N-glycoloylneuraminate, and meloside L. From the box plots in Figure 4, it can be seen intuitively that decanoylcarnitine had the highest level in healthy people among the four groups. However, the other four metabolites in healthy people had the lowest level. Decanoylcarnitine is a type of organic compound containing fatty acids and belongs to endogenous lipids. The change in the metabolic level of decanoylcarnitine occurs in many kinds of diseases, including ulcerative colitis, Crohn's disease, colorectal cancer, etc. [23, 24]. In our study, the plasm level of decanoylcarnitine decreased in three pulmonary nodule groups, which may be related to its increased demand and catabolic enhancement in the body with pulmonary nodule lesions. Klupczynska et al. [25] reported that patients with NSCLC had elevated carnitine levels and decreased amylcarnitine and propylcarnitine levels. Ni et al. [26] reported that the concentration of acylcarnitine in the plasm of lung cancer patients was significantly different from that of healthy people. Lim et al. [12] found that acylcarnitine was one of the main low-molecular metabolites that caused the difference in the metabolomics of lung tissues from various pathological types of lung cancer.

The major low-molecular metabolites between pulmonary metastatic carcinoma and healthy people were o-arachidonoyl ethanolamine, adrenoyl ethanolamide, tricin 7-diglucuronoside, and p-coumaroyl vitisin A. These four metabolites had the potential to be biomarkers for metastatic screening. From the FC values in Table 3, it can be found that the level of these four metabolites in PMC were much higher than that in HPG, and their contents in PMC were all more than five times that of HPG. Therefore, when these four substances were found to be significantly elevated in plasma samples from the screening population, it was suggested that attention should be paid to the possible presence of lung metastases. For such high-risk populations, further examinations, such as PET/CT, should be undertaken.

O-arachidonoyl ethanolamine is the first endogenous cannabinoid isolated and acts on the same receptor as tetrahydrocannabinol. Adrenoyl ethanolamide is a class of lipid compounds that naturally occur in animal and plant membranes and is a component of membrane-bound phospholipids. The area under the ROC curve of o-arachidonoyl ethanolamine and adrenoyl ethanolamide were 0.945 and 0.931, respectively, indicating that they can both distinguish lung metastatic cancer from healthy people. Tricin 7-diglucuronoside and p-coumaroyl vitisin A were also the major low-molecular metabolites between pulmonary metastatic carcinoma and healthy people. Tricin 7-diglucuronoside is a kind of organic compound called flavonoid. P-coumaroyl vitisin A belongs to a class of organic compound called coumarate. These aromatic compounds contain ester derivatives of coumaric acid. The AUC of ROC curves of these two metabolites were all greater than 0.95. The sensitivity and specificity of p-coumaroyl vitisin A for pulmonary metastatic cancer and healthy people were 0.875 and 0.979. However, the sensitivity and specificity of tricin 7-diglucuronoside were 0.813 and 1.000. It showed that these metabolites both had a strong diagnostic ability to distinguish pulmonary metastatic carcinoma from healthy people.

The common types of pulmonary nodules in clinical practice mainly include primary lung cancer, benign pulmonary nodule, and pulmonary metastatic carcinoma. Sometimes, the nature of pulmonary nodules cannot be accurately determined by chest CT and existing biomarkers, even with PET/CT examination. The exact nature of the nodule can only be determined by invasive methods such as puncture biopsy or surgical resection. In this study, these three common types of pulmonary nodules were taken as the research subject. First, an overall comparison was performed. It was found that there were significant differences in plasm metabolomics among them. The main low-molecular metabolites that caused the difference were anabasine, octanoylcarnitine, 2-methoxyestrone, retinol, decanoylcarnitine, calcitroic acid, glycogen, and austalide L.

In terms of differential diagnosis, pulmonary metastases were compared with two most common kinds of lung nodules, primary lung cancer (PLC) and benign pulmonary nodule (BPN). Tyrosine, indoleacrylic acid, and lysoPC (16 : 0) were selected from the comparison of PMC and BPN. Meanwhile, octanoylcarnitine, retinol, and decanoylcarnitine were discovered from PMC and PLC. These six metabolites could be used as biomarkers for differential diagnosis of pulmonary metastasis with other lung nodules. From the FC value of Table 2, we can see that the level of the first three metabolites in PMC was higher than that of BPN, indicating that when the three substances in plasma samples were higher, the nature of nodules is more inclined to lung metastasis, rather than benign pulmonary nodules. The levels of the latter three metabolites in PMC were much lower than those in PLC, suggesting that when plasma concentrations of these three substances decreased significantly, nodules were more likely to be lung metastases than primary lung cancer. Combining these results with the medical history and imaging findings, it would be easier to make an accurate diagnosis of the nature of the pulmonary nodule.

L-octanoylcarnitine is the physiological active form of octanoylcarnitine. Octanoylcarnitine can be detected in the middle-chain acyl-CoA dehydrogenase deficiency. Some studies had found that the metabolic level of octanoylcarnitine in the blood of patients with obesity and inflammatory bowel disease had been significantly changed [23, 27]. Retinol is also one of the main plasm low-molecular metabolites for differentiating pulmonary metastatic cancer from primary lung cancer. Its plasm content in patients with primary lung cancer is significantly higher than that of pulmonary metastatic cancer. Pamungkas et al. [28] reported the same conclusion with our study. Retinol (vitamin A) is a yellow fat-soluble antioxidant vitamin, which belongs to the family of retinoids. Taken in the form of precursors by the human body, retinol and its derivatives play a crucial role in the reproductive process, immune response, bone growth, epithelial growth and differentiation, and the metabolic function of the retina.

Tyrosine, indoleacrylic, acid and lysoPC (16 : 0) were the main low-molecular metabolites that caused the difference between pulmonary metastatic carcinoma and benign pulmonary nodules. The areas under the ROC curve of these metabolites were 0.852, 0.869, and 0.836, respectively, showing a good ability of differential diagnosis. Tyrosine is an essential amino acid that can cross the blood-brain barrier and a precursor of the neurotransmitters such as dopamine, norepinephrine, and epinephrine. This study found that the level of tyrosine in the plasm of patients with pulmonary metastatic carcinoma was approximately 1.7 times that of pulmonary benign nodule. Its sensitivity and specificity were 0.938 and 0.719, respectively. Klupczynska et al. [25] found that the concentration of tyrosine in the plasm of early non-small-cell lung cancer was significantly different from that of normal people, but this could not distinguish squamous cell carcinoma and adenocarcinoma. Hu et al. [29] conducted a metabolomics study of patients with advanced non-small-cell lung cancer who underwent microwave ablation and found that the plasm tyrosine content before treatment was significantly higher than that in the healthy control group, and after microwave ablation treatment, the level of tyrosine had dropped significantly.

Lysophosphatidylcholine (lysoPC or LPC) is present in a small amount in most tissues and is formed by the hydrolysis of phosphatidylcholine by the phospholipase A2. In this study, the plasm level of lysoPC (16 : 0) in patients with pulmonary metastatic carcinoma was significantly higher than that of benign pulmonary nodule, and its sensitivity and specificity were 0.938 and 0.687, showing its potential to the biomarker to distinguish them. Klupczynska et al. [15] carried out a metabolomic study on plasm samples from patients with stage I non-small-cell lung cancer and the noncancer control group and found that phospholipids containing choline were potential biomarkers for lung cancer. Besides, a model of seven metabolites composed of two fatty acid derivatives, four hemolytic phosphatidylcholine, and sphingolipids was established. Finally, they found that the metabolite with the strongest discrimination ability was lysoPC26 : 0 and lysoPC26 : 1. Yang et al. [30] reported a metabolomic research on malignant pleural effusion of advanced lung cancer and found that 25 ether lipids, including phosphatidylcholine (PC), lysophosphatidylcholine (LPC), and phosphatidylthanolamine (PE), were significantly downregulated in malignant pleural fluid, showing a good diagnostic potential. Ros-Mazurczyk et al. [31] found that the levels of lysoPC (18 : 2), lysoPC (18 : 1), and lysoPC (18 : 0) in patients with early-stage lung cancer were lower than those in healthy people.

## 5. Conclusions

From the perspective of clinical practice, this study aimed to find new potential biomarkers to improve lung metastases screening and differential diagnosis. Using the metabolomics method, we comprehensively studied the level change of low-molecular metabolites in plasm of pulmonary nodule lesion and healthy population. In particular, samples of pulmonary metastatic carcinoma were included in the research for the first time, and plasm metabolomics characteristics of pulmonary metastatic carcinoma were studied in depth. However, there are still some shortcomings in this study: First, the sample size of each group is small. Because metabolomics is the terminal stage of various biological changes, it is susceptible to various factors. The larger the sample size, the more reliable the results will be. Second, this research applies nontargeted metabolomics, so the low-molecular metabolites discovered can be further studied and identified by targeted metabolomics. Finally, although this study is based on clinical needs, the current research results are at the stage of basic research, and it is necessary to find a suitable way for clinical transformation to have greater significance.

Through this metabolomics study on plasm samples from pulmonary metastatic carcinoma, benign pulmonary nodule, primary lung cancer, and healthy people, it was found that the levels of some low-molecular metabolites were significantly different among the four groups. These major metabolites showed a good sensitivity and specificity, and they have the potential to become the biomarkers for the screening and differential diagnosis of lung metastases. This study laid a foundation for further research and clinical translation.

## Figures and Tables

**Table 1:** Comparison of general characteristics.

	PMC	BPN	PLC	HPG	*p* Value
No	16	32	80	48	

Age, *y* ± SD	55.2 ± 8.9	56.3 ± 8.9	55.0 ± 9.2	53.2 ± 9.2	0.317

Sex, *n* (%)					0.587
Male	12 (75.0)	24 (75.0)	57 (71.3)	35 (72.9)	
Female	4 (25.0)	8 (25.0)	23 (28.7)	13 (27.1)	

Smoking history, *n* (%)					0.069
Yes	10 (62.5%)	14 (43.8)	33 (41.3)	19 (39.6)	
No	6 (37.5%)	18 (56.2)	47 (58.7)	29 (60.4)	

Comorbidity, *n* (%)					0.345
Yes	7 (43.8)	13 (40.6)	27 (33.8)	15 (31.3)	
No	9 (56.2)	19 (59.4)	53 (66.2)	33 (68.2)	

Tumor site, *n* (%)					
LUL	4 (25.0)	4 (12.5)	21 (26.2)		
LLL	3 (18.8)	6 (18.8)	12 (15.0)		
RUL	7 (43.8)	5 (15.6)	23 (28.8)		
RML	0	1 (3.1)	4 (5.0)		
RLL	1 (6.2)	12 (37.5)	12 (15.0)		
Two or more	1 (6.2)	4 (12.5)	8 (10.0)		

Pathology types, *n* (%)					
Ad	13 (81.1)		65 (81.2)		
SC	0		10 (12.4)		
SCLC	0		2 (2.5)		
Carcinoid	0		1 (1.3)		
Carcinosarcoma	0		1 (1.3)		
LCLC	0		1 (1.3)		
ACC	1 (6.3)		0		
Others	2 (12.6)		0		

**Table 2:** The main metabolites in the comparisons of the pulmonary metastases with the other pulmonary nodule groups.

	Metabolites	Fold change	VIP	*p* Value	AUC of ROC	Critical point	Sensitivity	Specificity
PMC vs. BPN	Tyrosine	1.67	1.39	8.26E−5	0.852	1.71	0.938	0.719
	Indoleacrylic acid	1.76	1.40	3.57E−5	0.869	3.89	0.938	0.844
	LysoPC (16 : 0)	2.43	1.95	1.68E-4	0.836	0.53	0.938	0.687

PMC vs. PLC	Octanoylcarnitine	0.25	1.65	2.71E−9	0.973	6.51	0.888	0.938
	Retinol	0.08	2.25	8.53E−10	0.988	0.99	1.000	0.938
	Decanoylcarnitine	0.21	1.81	9.39E−9	0.956	3.01	0.875	1.000

**Table 3:** The main metabolites in the comparison of the pulmonary metastases with the healthy group.

	Metabolites	Fold change	VIP	*p* value (*E*)	AUC of ROC	Critical point	Sensitivity	Specificity
PMC vs. HPG	O-arachidonoyl ethanolamine	8.12	1.62	1.14–7	0.945	1.30	0.875	1.000
	Adrenoyl ethanolamide	7.56	1.54	2.86–7	0.931	43.77	0.813	1.000
	Tricin 7-diglucuronoside	5.45	1.46	4.82–8	0.958	5.64	0.813	1.000
	p-Coumaroyl vitisin A	6.03	1.51	3.39–8	0.964	2.13	0.875	0.979

**Table 4:** Feature parameters of comparisons among groups based on the OPLS-DA model.

	Four groups	PMC vs. HPG	Three groups	PMC vs. BPN	PMC vs. PLC
R2X	0.540	0.638	0.533	0.654	0.531
R2Y	0.624	0.983	0.704	0.956	0.976
Q2	0.598	0.939	0.625	0.787	0.949

## Data Availability

The datasets used and/or analyzed during the current study available from the corresponding author on reasonable request.
